# A versatile mathematical work-flow to explore how Cancer Stem Cell fate influences tumor progression

**DOI:** 10.1186/1752-0509-9-S3-S1

**Published:** 2015-06-01

**Authors:** Chiara Fornari, Gianfranco Balbo, Sami M Halawani, Omar Ba-Rukab, Ab Rahman Ahmad, Raffaele A Calogero, Francesca Cordero, Marco Beccuti

**Affiliations:** 1 Department of Computer Science, University of Torino, Torino, Italy; 2 Department of Molecular Biotechnology and Health Sciences, University of Torino, Torino, Italy; 3 Faculty of Computing and Information Technology, King Abdulaziz University, Jeddah, Kingdom of Saudi Arabia

**Keywords:** Non Linear Mathematical Models, Cancer Stem Cells, Parameter analysis

## Abstract

**Background:**

Nowadays multidisciplinary approaches combining mathematical models with experimental assays are becoming relevant for the study of biological systems. Indeed, in cancer research multidisciplinary approaches are successfully used to understand the crucial aspects implicated in tumor growth. In particular, the Cancer Stem Cell (CSC) biology represents an area particularly suited to be studied through multidisciplinary approaches, and modeling has significantly contributed to pinpoint the crucial aspects implicated in this theory.

More generally, to acquire new insights on a biological system it is necessary to have an accurate description of the phenomenon, such that making accurate predictions on its future behaviors becomes more likely. In this context, the identification of the parameters influencing model dynamics can be advantageous to increase model accuracy and to provide hints in designing wet experiments. Different techniques, ranging from statistical methods to analytical studies, have been developed. Their applications depend on case-specific aspects, such as the availability and quality of experimental data, and the dimension of the parameter space.

**Results:**

The study of a new model on the CSC-based tumor progression has been the motivation to design a new work-flow that helps to characterize possible system dynamics and to identify those parameters influencing such behaviors. In detail, we extended our recent model on CSC-dynamics creating a new system capable of describing tumor growth during the different stages of cancer progression. Indeed, tumor cells appear to progress through lineage stages like those of normal tissues, being their division auto-regulated by internal feedback mechanisms. These new features have introduced some non-linearities in the model, making it more difficult to be studied by solely analytical techniques. Our new work-flow, based on statistical methods, was used to identify the parameters which influence the tumor growth. The effectiveness of the presented work-flow was firstly verified on two well known models and then applied to investigate our extended CSC model.

**Conclusions:**

We propose a new work-flow to study in a practical and informative way complex systems, allowing an easy identification, interpretation, and visualization of the key model parameters. Our methodology is useful to investigate possible model behaviors and to establish factors driving model dynamics.

Analyzing our new CSC model guided by the proposed work-flow, we found that the deregulation of CSC asymmetric proliferation contributes to cancer initiation, in accordance with several experimental evidences. Specifically, model results indicated that the probability of CSC symmetric proliferation is responsible of a switching-like behavior which discriminates between tumorigenesis and unsustainable tumor growth.

## Background

The use of mathematical models to investigate biological systems is becoming progressively crucial to better understand their complex behaviors [1]. One of the remarkable results obtained through mathematical models is the suggestion of when and how cell fate determines the outcome of the phenomenon under study.

For instance, in cancers it is crucial to characterize when and how cells provide a balance between stem cells and daughter-cell lineages, since cell heterogeneity significantly contributes to tumor progression and maintenance. In this context, the acquisition of the tumor microenvironment concept has contributed to define a new generation of *hallmarks of cancer *[2], which extended the original ones [3] incrementing the complexity of the tumor biology. Specifically, different cell types make up tumor microenvironment which, on turn, preserves cell heterogeneity and provides regulations of cell individuality and cell collective functions. This continuous and finely tuned interplay can promote cancer outbreak, sustain tumor development and invasion, and provide niches for Cancer Stem Cells (CSCs) [4]. CSCs are defined as cells that possess the capacity to both self-renew and to generate the heterogeneous lineage of cancer cells comprising the tumor [5]. Moreover, they are considered cancer-promoters thanks to their ability of developing new tumors upon inoculating them into host mice [6]. Many evidences point out that CSCs drive tumor growth and evolution of several human cancers, such as lung [7], brain [8], colon cancers [9], etc.

In this paper we focus on CSC-based tumors, which have been largely investigated through approaches combining wet-lab experiments and mathematical techniques, as demonstrated by several papers [10-13]. CSC-tumors are hierarchically structured and characterized by different subpopulations of cells: CSCs, Progenitor Cells (PCs), and Terminal Cells (TCs). This heterogeneity influences both cancer progression and response to treatments, making fundamental the full understanding of the mechanisms underlying the CSC hierarchy [14]. In particular, the alternation of symmetrical vs. asymmetrical CSC division and the way in which feedback mechanisms - induced by microenvironmental changes - affect the tumor growth have been investigated in several papers [15-17]. However, it is not clear how the balance between the CSC asymmetric and symmetric division rates is maintained in order to preserve a constant level of CSCs in tumors and, at the same time, generate more differentiated cells.

To investigate this issue, we expanded our linear Ordinary Differential Equation (ODE) model on the initial phase of CSC-cancer growth [18] describing each stage of tumor progression. Our extended model is composed of CSCs, PCs, TCs and Dead Cells (DCs) subpopulations and it accounts for the tumor microenvironment effects, which can be modeled as mechanisms of auto growth limitation expressed by feedback controls on cell division. In our model tumor cells are assumed to progress through lineage stages like those of normal evolution, and a bounded cell division is introduced. These new features have introduced some non-linearities in the model, making it more difficult to be studied by solely analytical techniques. Thus, we have proposed a new analysis work-flow that helps to characterize system behaviors and to identify those parameters influencing such behaviors. The techniques that we have used range from statistical methods (as sensitivity analysis) to analytical studies (as bifurcation analysis). Their applications depend on case-specific aspects, such as the availability and quality of experimental data, and the dimension of the parameter space as well.

Therefore, the aim of this paper is twofold: (i) to extend our previous model on CSC-tumor growth - including more complex dynamics in the ODE system - and (ii) to provide a work-flow for the analysis of this class of models, using state of the art methodologies for sensitivity analysis [19].

Although the proposed methodology has been designed to study our tumor-growth model, it is not cancer specific. It could be applied to study dynamical systems in general, and to help in assessing parameter influences on ODE model dynamics. Moreover, the identification of key model parameters could also provide hints to design new experiments that might enhance the knowledge of the phenomenon under study.

## Materials and methods

### Population model

In Fornari *et al*. [18] we presented a system of ODEs focused on the description of the initial phase of CSC-tumor progression. The proposed model was linear and, consequently, its predictions relative to cell homeostasis were sensitive to small differences in parameter values. Indeed, to achieve homeostasis in any physical or biological system, feedback mechanisms are necessary to maintain stability in the face of infinitesimal parameter changes.

Here, we present an extension of our model, which accounts for stable cell homeostasis and considers cell subpopulation dynamics during the cancer growth. This has required to introduce: (i) a new cell subpopulation called Dead Cells (DCs) and (ii) a feedback control of cell number.

In details, the cell subpopulation dynamics are described through the following ODE system:

$$\begin{array}{c} \frac{dN_{CSC}}{dt}=P_{sy}\omega_{CSC}N_{CSC}+\gamma_{PC}N_{PC_{1}}-\eta_{1}N_{CSC}-\delta_{1}N_{CSC}\\ \frac{dN_{PC_{1}}}{dt}=\left(1-P_{sy}\right)\omega_{CSC}N_{CSC}-\omega_{PC}N_{PC_{1}}-\gamma_{PC}N_{PC_{1}}+\eta_{1}N_{CSC}-\eta_{2}N_{PC_{1}}-\delta_{2}N_{PC_{1}}\\ \frac{dN_{PC_{2}}}{dt}=2\omega_{PC}N_{PC_{1}}+\eta_{2}N_{PC}-\eta_{2}N_{PC_{1}}-\delta_{2}N_{PC_{1}}\\ \frac{dN_{TC}}{dt}=\eta_{3}N_{PC_{2}}-\delta_{3}N_{TC}\\ \frac{dN_{DC}}{dt}=\delta_{1}N_{CSC}+\delta_{2}\left(N_{PC_{1}}+N_{PC_{2}}\right)+\delta_{3}N_{TC}-\delta_{4}N_{DC} \end{array}$$

where *N_CSC _, N_PC _, N_TC _*and *N_DC _*are the total number of CSCs, PCs, TCs and DCs, respectively. Notice that we have modeled the PC subpopulation as made by two different levels: PC_1 _and PC_2_, i.e. $N_{PC}=N_{PC_{1}}+N_{PC_{2}}$. Possible cell behaviors are regulated by specific rates expressed through the following phenotypic parameters: *P_sy _*for the probability of symmetric CSC division; *ω_CSC _, ω_PC _*for CSC and PC_1 _proliferation; *η*_1_, *η*_2 _and *η*_3 _for CSC, PC_1 _and PC_2 _differentiation; *γ_PC _*for PC_1 _de-differentiation; *δ*_1_, *δ*_2 _and *δ*_3 _for CSC, PC, and TC death, while *δ*_4 _is used to determine the DC lysis.

**Figure 1:**
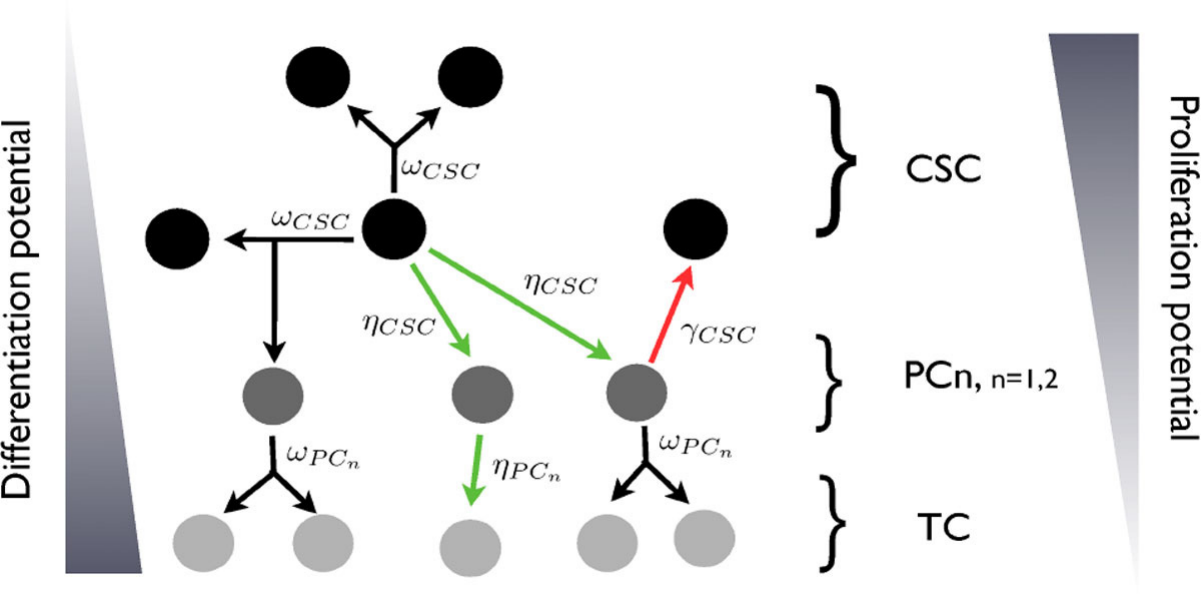
**CSC-based tumor model**. Simplified schematic representation of the cell subpopulation dynamics and interactions. The proliferative potential and differentiation degree are highlighted for each subpopulation: moving from the CSC compartment to the TC one, cells became more differentiated and lose their proliferative ability.

A graphical representation of the cellular dynamics described by system (1) is provided by Figure 1.

**Auto-growth limitation mechanism **Several papers [16,20] indicate that a computational model describing the growth of a CSCs-based tumor must take into account also the effects of the physical tumor microenvironment, which can be modeled as a feedback mechanism modulating phenotypic parameters. Therefore, to account for this cellular auto growth limitation, we introduced some feedback regulatory mechanisms to control division of CSCs and PC1s. In particular, their proliferation parameters *ω_CSC _*and *ω_PC _*were defined as:

(2) $$\omega_{CSC}\to \frac{\omega_{CSC}}{1+h_{CSC}N_{TC}},\;\omega_{PC}\to \frac{\omega_{PC}}{1+h_{PC}N_{TC}},$$

where *h_CSC _*and *h_PC _*correspond to the feedback intensities. Namely, CSC and PC_1 _proliferation rates now depend on the TC number, which is in agreement with the knowledge that the growth and progression of cancer cells depend not only on their intrinsic malignant potential, but also on a mutual and continuous dialog among them and the tumor microenvironment [4]. Indeed, the growth conditions of CSCs are influenced by blood supplies, growth factor and, in particular, local cell types. Considering several experimental observations [16,15] we parameterize this knowledge by specifying with equations (2) a bounded cell division regulated by the density of TC subpopulation.

Finally, note that the introduction of the auto growth mechanism makes the ODE system (1) non linear.

### Methodology

In this section we describe the methodology developed to study our complex model showing how it is sufficiently general and powerful to be effectively applied when the presence of uncertainties in experimental data makes the analysis of biological models extremely complicated.

The general outline of our methodology can be summarized by the following three main phases which will then be subsequently commented in detail:

1 input/output characterization

1.1 sampling of model inputs through the Latin Hypercube Sampling (LHS) technique;

1.2 investigation of possible model behaviors deriving from different parameter samples, i.e. creation of model outputs;

2 key parameter identification

2.1 evaluation of Partial Rank Correlation Coefficients (PRCCs) between model parameters (inputs) and model behaviors (outputs), at different time points;

2.2 identification of model parameters and time points which need to be carefully studied;

3 key parameter analysis

3.1 colored visualization of model outputs (i.e. variable values over time) with respect to the values of *"key" *parameters identified during the previous step;

3.2 creation of scatter plots representing model outputs (at designated time points) versus parameter values, colored in accordance with key parameter values;

3.3 accurate characterization of the role of the selected parameters through analytical techniques (as bifurcation analysis).

The application of the LHS method and PRCC analysis may be performed starting from Matlab functions described in [19], enhanced to extend their analysis capabilities of specific parameters by plotting graphs colored according to key parameter values. Bifurcation analysis may be performed using the graphical Matlab package MatCont.

#### Input/output characterization

**Figure 2:**
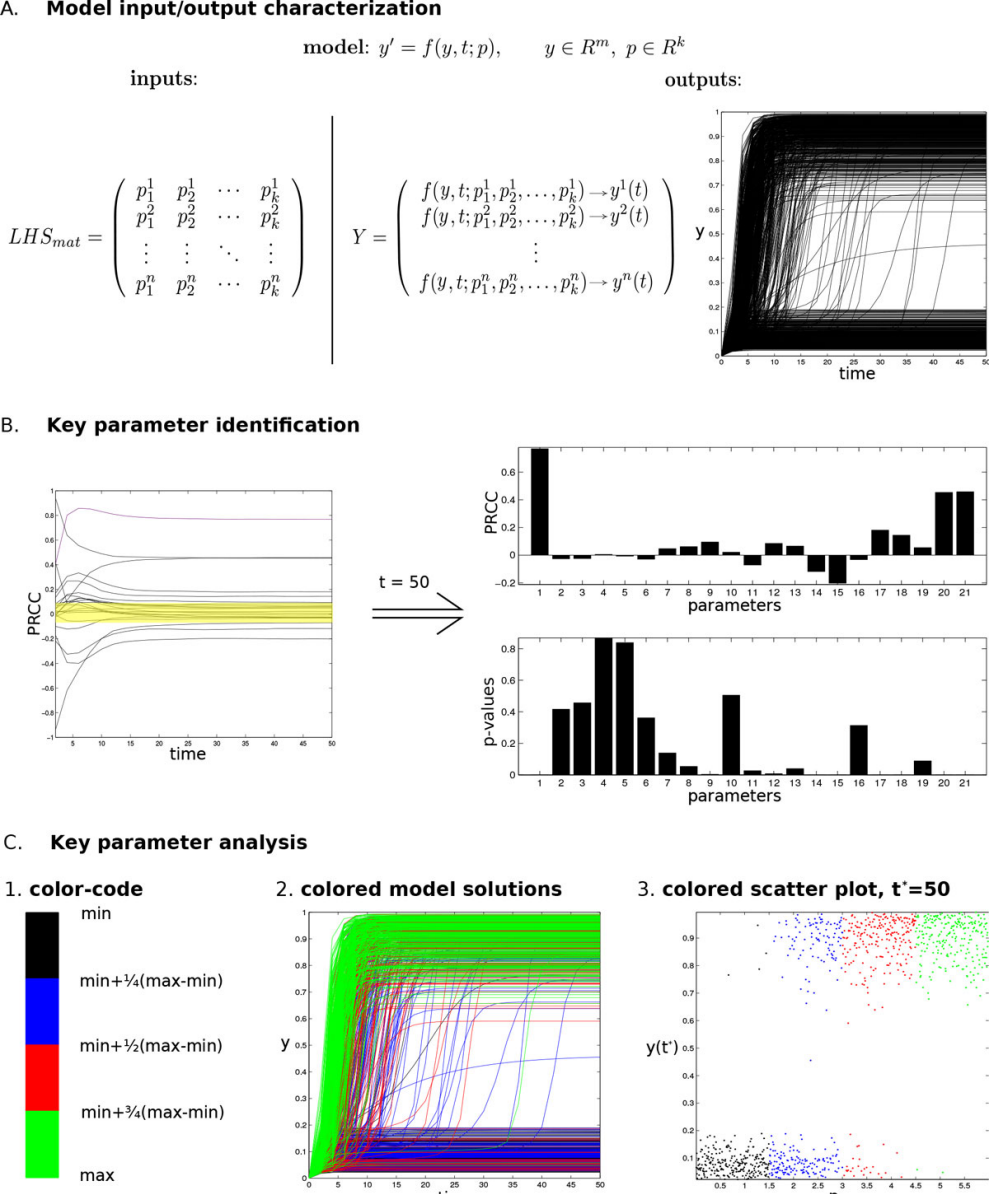
**Sketch of the methodology**. The proposed methodology consists of three main phases: (i) input/ouput characterization, (ii) key parameter identification, and (iii) key parameter analysis. Panels provide a general outline of each phase through a graphical representation of the proposed work-flow. Panel A - *input/output characterization*: model inputs are sampled (LHS technique) and then, starting from these values, model solutions are evaluated over time thus investigating possible model behaviors. Panel B - *key parameter identification*: monotonic relationships among model inputs and outputs are revealed with PRCC analysis over time, thus identifying key model parameters and time points where to expand the analysis. For each selected time, PRCCs and their significance (p-values) are provided. The yellow area shows non significant PRCCs. Panel C - *key parameter analysis*: C1. values of key parameters are partitioned into *r *equal intervals, and each interval is associated with a different color, thus designing a color-code. C2: model traces (i.e. time dependent outputs) are then colored following the color-code, thus highlighting the role of the selected parameter. Notice how the high concentrations of model outputs is mainly characterized by green lines, i.e. by high values of the key parameter. C3: relations among model outputs and key parameters are further emphasized by scatter plots (at a fixed time point), colored following the color-code.

The temporal behavior of a deterministic model (i.e. model output) is completely determined by its structure and by the values of its parameters (i.e. model input) [21]. Unfortunately, due to their intrinsic biological variability, parameter values are usually not completely determined and often measured with low accuracy, making them difficult to be estimated. Therefore, it is crucial to have techniques which allow to explore model behaviors resulting by changes of parameter values, such that it would be possible to investigate the uncertainty of model outputs deriving from the uncertainty in parameter inputs (Uncertainty Analysis - UA) [22]. Monte Carlo (MC) methods, which are based on probabilistic sampling procedures, are often used to develop a mapping from model inputs to model outputs and to perform UA. More precisely, in a MC simulation multiple model evaluations are performed using random numbers to sample from probabilistic distributions of model inputs. Many papers are published in the literature to discuss these approaches, and several sampling strategies are available and immediately implementable [23,24]. One of the most used MC method is the Latin Hypercube Sampling (LHS), which is a stratified sampling without replacement technique that generates sets of parameter values from a multidimensional distribution [25,26]. More precisely, for each model parameter the sampling process is driven by a probability density function (pdf), which frequently corresponds to the uniform distribution. The uniform pdf is adopted in the following two main cases: (i) when it is known only a putative range for the parameter values; (ii) when it is useful to have a homogeneously spread set of parameter values within an interval of interest. Instead, when some knowledge suggesting the expected value of a parameter is available, a normal distribution can be used. For both distributions, a set of baseline parameter values is required to start the sampling process. LHS provides a good coverage of each parameter variability by dividing each parameter range into *n *(sample size) equal-probability subintervals, which are sampled exactly once. Notice that, there is no a priori exact rule for determining *n*, which - in general - should be greater than *k *+ 1 (*k *is the number of model parameters). LHS method also assumes that the sampling is performed independently for each parameter. After this process, a *n × k *matrix (LHS matrix) is generated, containing the *n *sampled sets of values for all the *k *model parameters, as showed by Figure 2, panel A.

Starting from these input values, each row of the LHS matrix is used as an input to numerically integrate the system over the time interval *T *= [*t_ini_, t_end_*], and thus producing *n *time-dependent model solutions. The time points are selected to homogeneously cover the time interval of interest. If specific time subintervals are known to be relevant, they can be investigated increasing the number of time points in these subintervals of *T *. Model outputs (matrix Y) are then collected for each experiment considering the different time points of *T *, such that model temporal behaviors (i.e. model traces) can be derived. Specifically, a plot providing a graphical representation of these time-dependent traces is produced for each parameter combination, as sketched in Figure 2, panel A.

#### Key parameter identification

To identify critical inputs and to quantify how they impact model outcomes, Partial Rank Correlation Coefficients (PRCCs) between model parameters (LHS matrix) and model outputs (Y matrix) are evaluated on the interval *T *. Indeed, PRCC measures monotonic relationships between outputs and inputs and it provides a measure of monotonicity after the removal of the linear effects of all but one variable [26]. Considering multiple time points, PRCCs between model variables and model parameters are evaluated and plotted, as shown in Figure 2, panel B. PRCC analysis is done in an exploratory way to identify any significant relationships throughout the entire time course, and to point out whether correlations occur either over the entire time interval, or at specific time points. Since even small correlations may be significant, statistical tests assessing if the PRCCs are different from zero are also performed [19]. Details on the indexes that can be used are provided in [27].

PRCC analysis and corresponding significance tests are then used to identify key model parameters and to select time points which need an in-depth study. Specifically, among all model parameters, only those with *high *and *significant *PRCCs for all variables are further investigated, and the analysis starts from those parameters having the *highest *PRCCs values. More precisely, PRCC values close to 1 (-1) identify positive (negative) monotone relationships between inputs and outputs, by definition. In addition, significance tests allow to discover those correlations that are important, despite having relatively *small *PRCC values, namely those correlations that have significant p-values. In this phase of the study it is crucial the role of the analyst who, considering the PRCCs and p-values, decides on which parameters focus the study, and whereas focus it on the whole time interval or on specific time points. A general criterion to guide this process is difficult to be defined, since it strongly depends on the peculiarities of the model under investigation.

#### Key parameter analysis

After the identification of the relevant model parameters, a detailed analysis of their effects on model outputs is performed as concluding step of our methodology. For the sake of simplicity we will describe this process considering only one *relevant *parameter, that we will call *p*_1_. The proposed approach can however be easily extended to a set of relevant parameters, {*p*_1_*, ..., p_k _*}, reproducing such analysis *k *times and then cross-analyzing the results. An example of cross-analysis is provided in our second case study (i.e. the apoptosis model), where two relevant parameters have been identified and their cross-analysis have been necessary to explain model bistability.

The variation range of parameter *p*_1 _is divided into *r *subintervals, such that the *p*_1_'s possible values are partitioned into *r *levels. The choice of *r *depends on several factors, such as the variation range of the parameter and its expected qualitative behavior. Similarly to the adaptive numerical methods, an *adaptive *partition could be defined with a varying *r*, such that the most a parameter is critical in a region, the smallest *r *is set to cover that region. Then, as shown in Figure 2 - panel C1, a specific color is assigned to each of these levels and model traces are colored accordingly to this classification. Specifically, each model trace is colored in accordance with the *p*_1 _value used for its computations, i.e. each trace has the same color of the subinterval whom its *p*_1 _value belongs to; see Figure 2, panel C2. This new representation turns out to be very effective in showing how particular model behaviors, such as switches, bistabilities, etc., are related to the *p*_1_'s variation. This visualization of model behaviors can thus be considered as a preliminary, but effective, analysis of the role that *p*_1 _plays in the global model dynamics.

The subsequent step consists in expanding the analysis at selected *interesting *time points, i.e. at those points which are known to be crucial for the problem under investigation, or which have been previously detected as relevant in PRCC analysis. Scatter plots of model outputs versus *p*_1 _values (or other relevant parameters, or combinations of them) are evaluated and colored in accordance with the parameter variation range. As depicted in Figure 2, panel C3, colored scatter plots enable a simple and direct visual detection of correlations between model inputs and outputs, emphasizing the role of *p*_1_.

Let us note that when few parameters are identified as relevant for model dynamics, the previous analysis is performed for each of them trying to discover multiple dependencies.

Finally, after having identified in a qualitative manner the key model parameters, an analytic study is performed to obtain an accurate characterization of the actual role of these parameters using more sophisticated mathematical techniques, such as bifurcation analysis.

## Results

Before using our methodology to explore the dynamics of the extended CSC-tumor model (1), we verified the effectiveness of this approach on two well-known and experimentally validated models by Tyson *et al *[28]. These models - that describe the oestrogen signalling network in breast epithelial cells - have been widely studied by Tyson and coworkers, exhibit bistable switches, and are among the reference models in mathematical biology.

The following sections report the results of our numerical experiments, whose settings are summarized in Table 1. In all cases we performed LHS using uniform distributions to have spread and un-biased samples of parameter values. In particular, when referred to models proposed by Tyson, this choice allowed to test our approach in a "blind" manner without taking advantage of knowledge that has already been published in the literature. Moreover, we partitioned all parameter intervals using *r *= 4 equal subintervals to start from informative divisions which were also easy to manage. In all cases this choice (i.e. *r *= 4) provided sufficiently confined but explanatory partitions, which did not require further refinements. The general color code that we used in all cases is: (i) *black *for values in $I_{1}=\left[min,\frac{1}{4}\left(max-min\right)+min\right]$, (ii) *blue *for values in $I_{2}=\left[\frac{1}{4}\left(max-min\right)+min,\frac{1}{2}\left(max-min\right)+min\right]$, (iii) *red *for values in $I_{3}=\left[\frac{1}{2}\left(max-min\right)+min,\frac{3}{4}\left(max-min\right)+min\right]$, and (iv) *green *for values in $\begin{array}{c} I_{4}=\left[\frac{3}{4}\left(max-min\right)+min,max\right] \end{array}$.

**Table 1:** Settings of numerical experiments.

Model	*n*	pdf	var (%)	baseline values	*T*	*t*	*r*
cell cycle	1000	unif	*±*25	[28]	[0, 50]	50	4
apoptosis	1000	unif	*±*30	[28]	[0, 500]	500	4
population	1000	unif	*±*50	Additional File 10	200	[0, 200]	4

### Cell cycle model

In [28], cell cycle is modeled describing the interactions among a set of key proteins which control the G1-to-S phase transition in mammalian cells, namely RetinoBlastoma protein (RB), Cyclin D (CycD), the E2F family of transcription factors (E2F), and Cyclin E (CycE). The cell choice between quiescience and proliferation is controlled by a bistable switch characterized by an OFF state (quiescient cell arrested in G1 phase) and an ON state (proliferating cell progressing through S, G2 and M phase). Analyzing this model, the authors characterized the role of each protein in maintaining alternation between the two stable steady states.

#### Input/output characterization

**Figure 3:**
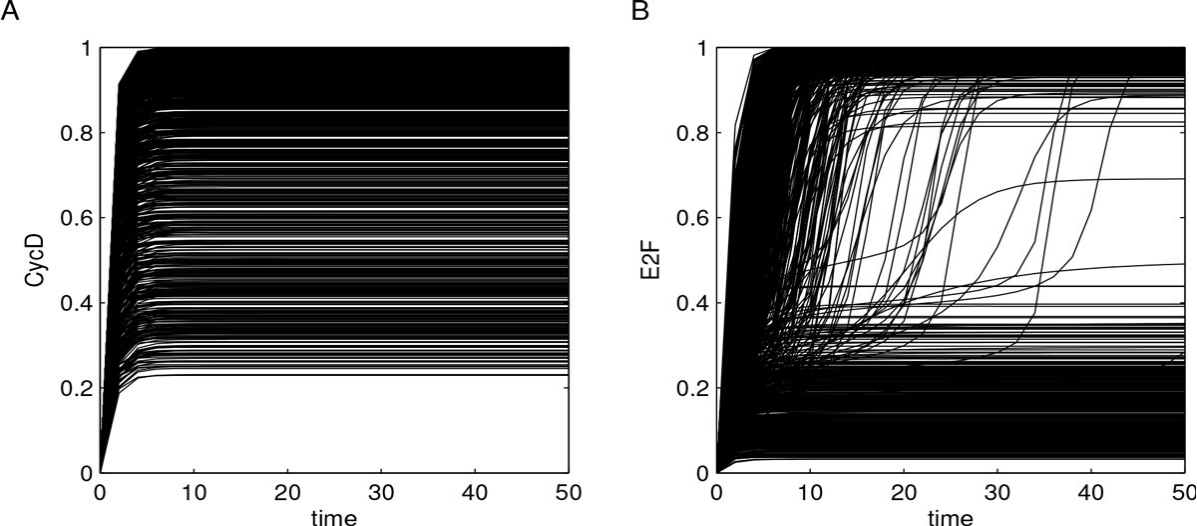
**Outputs of cell cycle model**. Model solutions were calculated for each parameter combination over the time interval *T *= [0, 50], and are here reported for CycD (panel A) and E2F (panel B). The bimodal behavior of E2F results evident from these experiments.

Parameter values were sampled by means of LHS, starting from values defined in [28] and reported in Additional File 1 (baseline parameters). *n *= 1000 parameter combinations were generated using uniform distributions, whose minimum and maximum values were determined using baseline parameter values augmented with *±*25% to sample within neighborhoods of the values reported in [28]. We explored the parameter space using uniform distributions to work with spread samples of parameter values and to infer results that were not influenced by those reported in [28]. Model solutions were then calculated for each parameter combination over the same time interval *T *= [0, 50] analyzed in [28], as reported in Figure 3 for CycD and E2F and in Additional File 2 for CycE and RB. From this preliminary investigation the bimodal behavior of E2F and CycE resulted evident, suggesting the presence of interesting dynamics.

#### Key parameter identification

**Figure 4:**
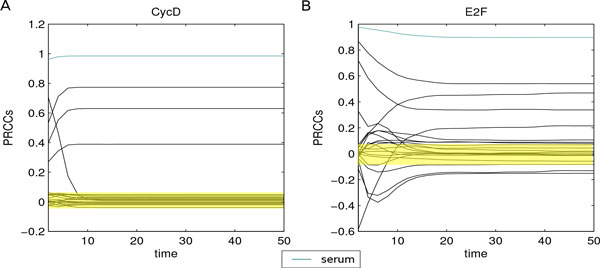
**PRCC analysis of cell cycle model**. PRCCs of input/output data revealed serum as the key parameter. Results relative to CycD and E2F during the whole time interval are reported in panels A and B, respectively. Yellow areas represent the zones of non-significant PRCC values, while blue lines correspond to serum PRCC values. The strong positive monotonic relationship between serum and model outputs is remarked, being blue lines close to one in the whole interval and *isolated *from the other PRCC

A PRCC analysis of input/output data was performed to identify key model parameters. Serum concentration resulted to be the most relevant one, since it has the strongest correlation (close to 1) with all model variables during the whole time interval (biological experiments on how serum concentration affects cell cycle are reported in [28]). Specifically, the PRCC of serum concentration over time appears remarkably different from the PRCCs of other outputs, as can be noted in Figures 4 andAdditional File 3 which report the estimated monotonic relationships between inputs and outputs for CycD, E2F and CycE, RB respectively. Significance tests of the correlation among serum and model variables confirmed these results with p-values *<*0.01.

#### Key parameter analysis

**Figure 5:**
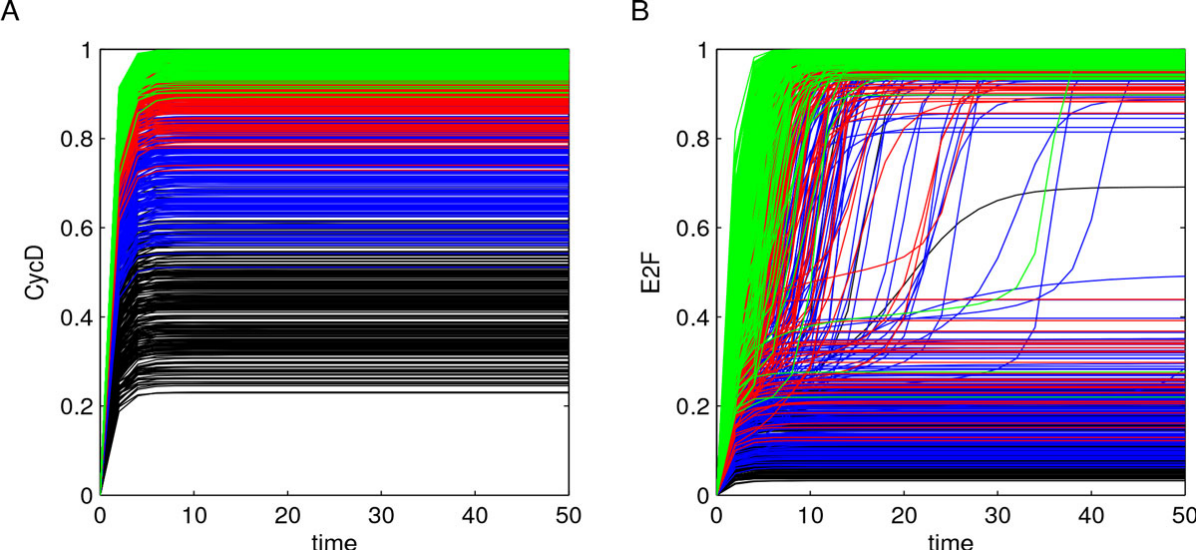
**Serum variation influences cell cycle dynamics**. A color-code identical to that of Figure 2 panel C1 was defined for serum variation (see Table 2), and model outputs were then consequently colored. Panels A and B report the colored visualizations of CycD and E2F, respectively. CycD levels increase with serum concentration: colors are well clustered and stratified in the order expressing a serum increase (black-blue-red-green). E2F distribution, instead, exhibits a bimodal dependence on serum concentration: low E2F state is mainly characterized by black and blue lines (low serum concentration), while the high state corresponds to green lines (high serum concentration).

We further investigated the model focusing on serum concentration to characterize how its variation influences model dynamics. Serum concentrations were divided into the *r *= 4 intervals of the same amplitude reported in Table 2, and a specific color was assigned to each subinterval (level), as shown in Figure 2, panel C1. Plots representing model traces were hence colored using this color-code, as reported in Figure 5 for E2F and CycD and in Additional File 4 for CycE and RB. CycD levels increase smoothly with serum concentrations, while the E2F distribution exhibits a bimodal dependence on serum levels. Indeed, in Figure 5 panel A, colors are very well clustered, being the traces stratified in accordance with serum concentrations (black-blue-red-green). In Figure 5, panel B, traces follow the same stratification order, but clusters are less evident. Anyhow, the E2F bistable behavior is well explained by variation in serum concentrations, since the low E2F state (below 0.5) is mainly characterized by low serum concentration values (black and blue lines), while the high state (over 0.5) corresponds to high (serum) concentrations (green lines).

**Table 2:** Color codes.

Model	parameter	I_1 _(black)	I_2 _(blue)	I_3 _(red)	I_4 _(green)
cell cycle	serum	[0.01, 1.50]	(1.50, 3.00]	(3.00, 4.49]	(4.49, 5.99]
apoptosis	stress	[0.01, 0.25]	(0.25, 0.50]	(0.50, 0.75]	(0.75, 0.99]
apoptosis	BCL2T	[56.00, 68.00]	(68.00, 80.00]	(80.00, 91.99]	(91.99, 103.99]
population	*P_sy_*	[0.00, 0.25]	(0.25, 0.50]	(0.50, 0.75]	(0.75, 1.00]

The variation ranges of key parameters are partitioned into *r *= 4 equal subintervals, whose values are reported together with their colors.

**Figure 6:**
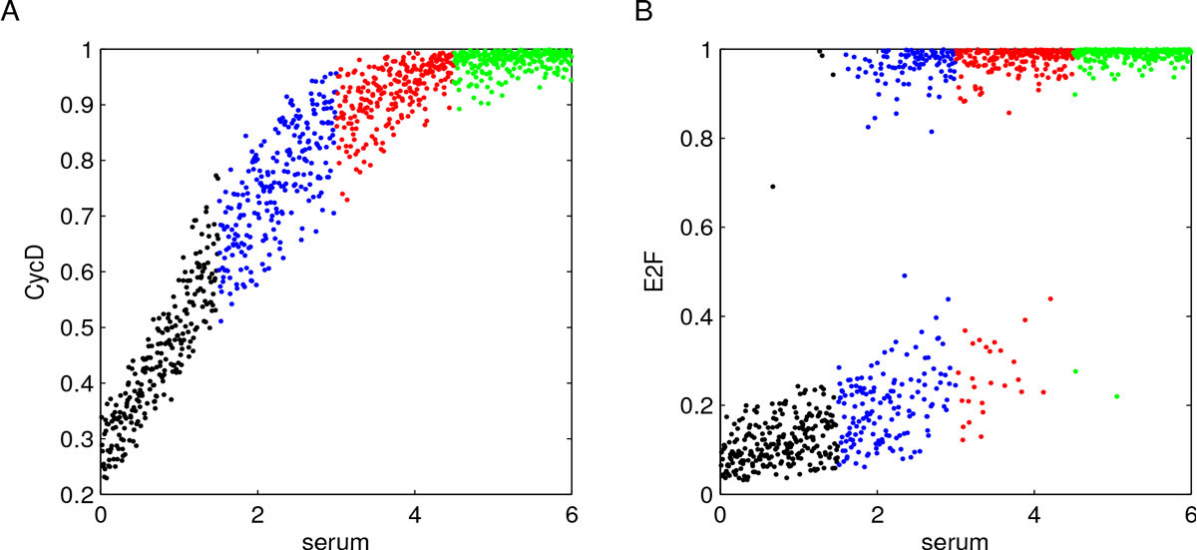
**Scatter plots of cell cycle and serum variation**. Colored scatter plots of model variables at time *t *= 50 versus serum variation were produced, and are here reported for CycD and E2F in panles A and B respectively. This graphs show CycD and E2F configurations at equilibrium, further emphasizing the role of serum variation. In panel A, CycD levels increase with serum concentration making evident the positive monotonic relationship between serum and CycD. In panel B, instead, the low E2F state is characterized by black-blue points (low serum), while the high state by red-green points (high serum), thus revealing that E2F distribution exhibits bimodal dependence on serum concentration.

To enhance this analysis we focused on scatter plots of model variables at the final time of our experiments (i.e. *t *= 50) versus serum variation, coloring the plot points in accordance with serum levels. This graphs showed model configurations at equilibrium, further emphasizing the model evolutions as well as the role of serum in cell cycle. Figure 6, panel A, shows the positive monotonic relationship between serum and CycD. Instead, in Figure 6, panel B, it is very clear that E2F distribution exhibits a bimodal dependence on serum concentrations: the *low *state (below 0.5) is characterized by low serum levels (black and blue points), while the *high *state (over 0.5) by high serum levels (green points). Similar results can be found in Additional File 5, where scatter plots of CycE and RB versus serum values, at *t *= 50, are reported. Both proteins have a monotonic relationship with serum concentrations, and the one of CycE also bimodal.

Despite the use of completely different approaches, the results obtained from this first case study reproduced exactly those presented by Tyson *et al*. in [28]. This agreement is a preliminary validation of our work-flow and thus supports the usage of our methodology.

### Apoptosis model

From the same paper [28], we selected a second model to validate our methodology. This second case study concerns apoptosis in mammalian cells and it is modeled by Tyson *et al*. through a bistable system. The irrevocable commitment to apotosis reaches a one-way decision point in which pro-death and pro-survival signals are processed determining cell fate. In [28], the authors focused on interactions among proteins BAXm, BCL2, and BH3, which are responsible of the bistable and irreversible switch governing apoptosis. Specifically, the switch is OFF or ON depending on the balance among BAX, BCL2 (*brake*) and BH3 (*accelerator *): in the OFF state BAX is inactivated by binding to BCL2, while in the ON state BAX is active since BCL2 is displaced due to BH3 accumulation.

#### Input/output characterization

**Figure 7:**
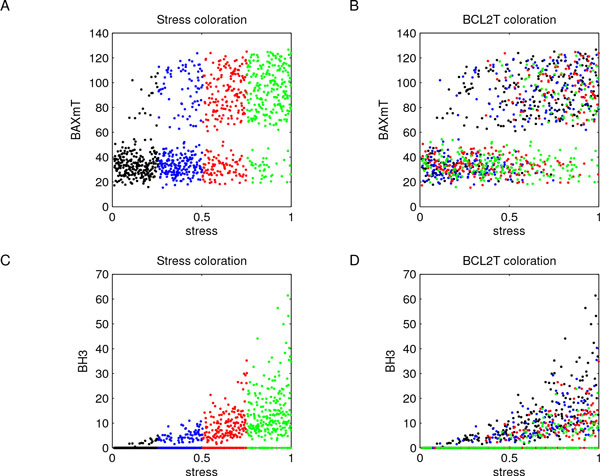
**Stress and BCL2T concentrations control cell apoptosis**. A color-code identical to that of Figure 2 panel C1 was assigned to both stress and BCL2T variations; see Table 2 for the subinterval definition. Scatter plots of variables versus stress variations at time *t *= 500 were then evaluated, and they are here reported for BAXmT (panels A and B) and BH3 (panels C and D), colored for both color-codes. Specifically, panels A and C are colored for stress, while panels B and D are colored for BCL2T. Both BAXmT and BH3 can be found in two different configurations, depending on the concentrations of stress and BCL2T.

As in the previous case study, we began our analysis characterizing model input/output. Specifically, starting from the parameter values reported in Additional File 6 we created a sample of *n *= 1000 parameter combinations performing LHS with uniform distributions. In accordance with [28], stress values were sampled within the interval 0[1], while the range of other distributions were fixed using baseline parameter values augmented with *±*30% to work with spread samples of parameter such that our results were not influenced by those reported in [28]. As before, LHS settings were fixed to homogeneously sample parameter values within neighborhoods of the baseline parameter values defined in [28]. Then, 1000 model solutions were computed - i.e. one for each parameter combination - and this made evident the switching-like behavior of the system. In particular, considering the time interval *T *= [0, 500] reported in [28], it resulted that BAXmT and BH3 can stabilize on two (stable) concentrations: low/high values. Additional File 7 reports the temporal behaviors of all the involved proteins over time, while Additional File 8 shows the same behaviors coloured for both stress and BCL2T. BAXmT and BH3 concentrations at equilibrium (*t *= 500) are showed in Figure 7 as scatter plots.

#### Key parameter identification & analysis

Contrary to the cell cycle model where we found only one relevant parameter, PRCC analysis on apoptosis model revealed two putative key parameters, namely stress and BCL2T (total BCL2 concentration). As reported in Additional File 9, they both have high PRCC values (*|*1*| *and *|*0.8*|*, respectively) in the whole time interval [0, 500]. Significance tests resulted in p-values *<*0.01, so that we expected them both to be related with the bistable apoptosis switch. We defined a color-range made of *r *= 4 variations for both stress and BCL2T (see Table 2 for values), and model traces were then colored and analyzed twice (i.e. one for each parameter). Specifically, as shown in Additional File 8, traces became clustered by colors and color sequences followed orders describing the increase/decrease of the two parameters. By means of this approach we were able to *qualitatively *detect the monotonic relationships between stress and BH3, stress and BCL2, BCL2T and BH3. We also pointed out how model bistability depends on variations in parameter values, being high/low states of BAXmT and BH3 characterized by specific parameter concentrations (i.e. colors). To further characterize these dependencies it was necessary to cross-analyze the correlations among variables (BAXmT, BH3) and parameters (stress, BCL2T), since they were not sufficient to explain model bistability when considered separately. In detail, we analyzed scatter plots of variables versus stress variations at the end time of our numerical experiments (i.e. *t *= 500), and we colored the resulting plots twice: one applying stress color-code, the other one focusing on BCL2T variations. As shown by Figure 7 (panels A and B), BAXmT can be found in two concentrations (high and low), depending on the amount of both stress and BCL2T. Indeed, when cells are in *extremely *high or low stress conditions, BAXmT concentration is mainly in one configuration; see the points in the areas at the left or right margins of the plots which are mainly concentrated below or over 60. Specifically, as reported in Figure 7, panel A, very low stress values induce low concentrations of BAXmT (black points below 60), while highly stressed cells have high BAXmT concentrations (green points above 60). Instead, for intermediate values of stress (blue and red points), BAXmT has two stable steady states: a low and a high one (below and over 60, respectively). As shown in Figure 7, panel B, these configurations are mainly characterized by the concentration of BCL2T. When BCL2T is high, BAXmT is in the low configuration no matter how much stress is affecting the system, as resulted from the the green points below 60 in panel B.

Similarly, BH3 may evolve into two states: (i) a *zero *one in which the protein is almost absent, and (ii) a positive one, in which the protein concentration increases as stress does (see Figure 7, panels C and D). Let us note that, while the positive state is sensitive to stress variation, the zero one is independent of the same fluctuation. Indeed, as it is evident from Figure 7 panel C, BH3 may end in the zero-configuration for each stress concentration. In fact, what really defines this zero-state is the high concentration of BCL2T, as evident from the green points in Figure 7, panel D.

Summarizing, following our methodology we were able to detect bistabilities in the apoptosis model and to characterize different model evolutions in terms of parameter changes, reaching conclusions similar to those discussed in [28].

### Application to our case study

The aim of the previous part of the study was to test (validate) the proposed methodology on well-known case studies. Having gained confidence in the approach, we applied the method to our population model (1) to explore the phenotypic characteristics inherent to CSCs on tumor growth. Indeed, system (1) is governed by a number of cellular behaviors which are difficult to measure with biological experiments due to their high variability. The proposed approach helps to understand better how the system functions and which phenotype parameters mostly influence it.

#### Model input/output characterization

Following the indications of the first phase of our methodology, parameter values (model inputs) were generated with the LHS technique and time-dependent subpopulation behaviors (model outputs) were evaluated for each sampled parameter combination.

As described in *Materials and Methods*, the LHS technique needs a set of baseline parameter values to start from. These rates and the initial cell concentrations were tuned starting from values found in the literature [18,29,30] and provided by experimental evidences. Moreover, initial conditions were fixed to reproduce specific subpopulation proportions: TCs were set as the largest subpopulation - as they represent the main part of the tumor mass - and CSCs as the smallest one [18]. Parameters were then retrieved by tuning system (1) to reproduce the growth trend of tumor mass observed in BALB/c mice after a subcutaneous injection of 10^5 ^cancer cells [31]. Baseline parameter values and model initial conditions are reported in *Supplementary Materials *as Additional File 10.

**Figure 8:**
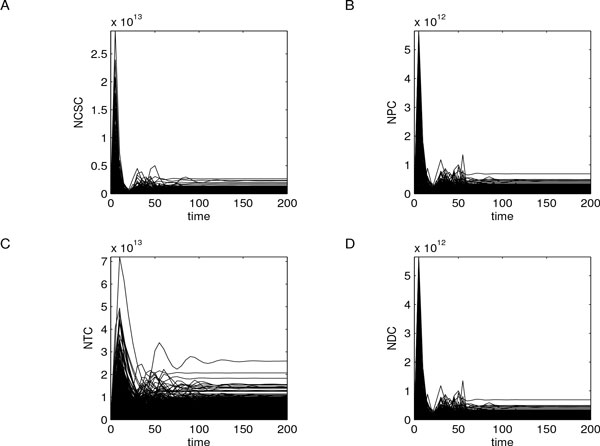
**Cell subpopulations dynamics**. System (1) was solved 1000 times over the interval *T *= [0, 200] using each sampled parameter combination. Results are here reported for each cell subpopulation: panel A refers to CSCs, panel B to PCs, panel C to TCs, and panel D to DCs. It resulted that, after a first oscillatory phase, subpopulations reach equilibrium values. However, the wide variation range of output data could make less evident some interesting subpopulation behaviors.

A sample of *n *= 1000 parameter combinations was generated through LHS using uniform distributions, whose sampling intervals were evaluated starting from the baseline values reported in Additional File 10 augmented with *±*50%. The choice of these variation ranges was due to the lack of experimental data needed to unequivocally estimate the parameter values; moreover, using 1000 combinations of model parameters allowed us to widely explore model behaviors. Indeed, system (1) was solved 1000 times via numerical integration on the time interval *T *= [0, 200], using in each run a different set of parameter values. Figure 8 reports these numerical experiments and shows how, after a first oscillatory phase, the different subpopulations reach equilibrium values. Notice that output data have a wide range of variation - in term of the difference between their maximum and minimum values - which could hide interesting subpopulation behaviors.

#### Key parameter identification

Correlations between model inputs and model outputs were evaluated over the whole time interval [0, 200] to assess which phenotype parameters mostly influence subpopulation dynamics in terms of monotonic relationships.

**Figure 9:**
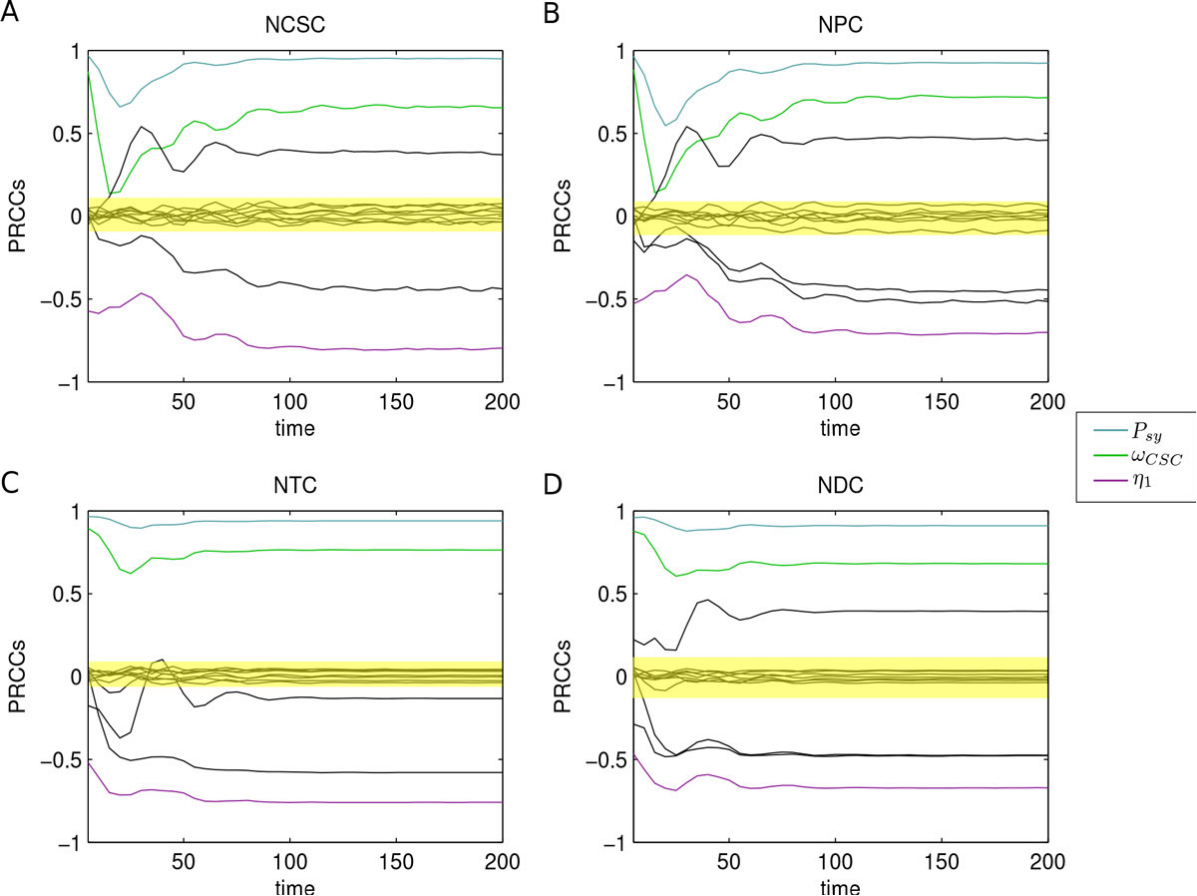
**PRCC analysis of CSC-based tumor model**. PRCC analysis revealed *P_sy _*(blue line), *ω_CSC _*(green line), and *η*_1 _(violet line) as the key model parameters. They have high and significant PRCC values on the whole time interval and with respect to all model variables, as reported in panel A for CSCs, panel B for PCs, panel C for TCs, and panel D for DCs. Among these parameters, *P_sy _*is the one with the highest PRCCs, and was hence selected to drive the analysis. Yellow areas represent the zones of non-significant PRCC values.

We found that the overall model dynamics mainly correlate with a small set of parameters related to changes in CSC subpopulation. In particular, CSC symmetric proliferation probability (*P_sy _*) resulted correlated with all the output variables, for the entire time interval. *P_sy _*has the highest PRCC values (almost 1) with respect to each model variable, and with significant p-values *<*0.01. Other interesting correlations were also found for CSC proliferation (*ω_CSC _*) and CSC differentiation (*η*_1_) parameters (almost 0.8 and -0.8, respectively, with significant p-values *<*0.01). Figure 9 summarizes these results showing the key parameters with colored lines and highlighting which monotone relationships they have with model variables.

We proceeded with the study firstly investigating the role of *P_sy _*and, then, exploring those of *ω_CSC _*and *η*_1_.

#### Key parameter analysis

We firstly focused our study on *P_sy _*, fixing *r *= 4; see Table 2 for the partition of *P_sy _*values. Model traces were colored in accordance with their *P_sy _*values to detect how *P_sy _*variation affects model dynamics. Output data were also expressed using a logarithmic scale to reduce their wide range to a more manageable size, thus allowing to pinpoint some interesting behaviors that have been subsequently studied in detail.

**Figure 10:**
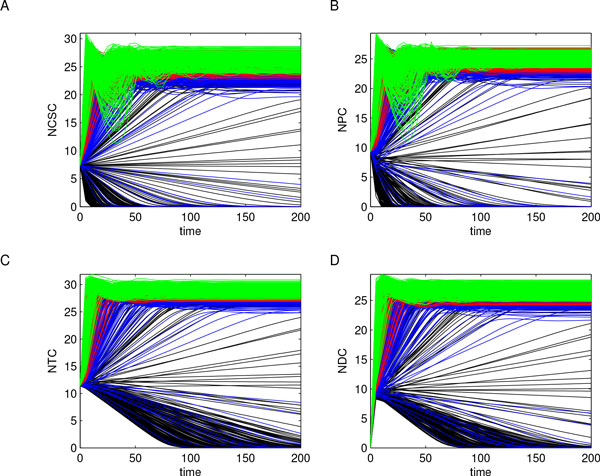
***P_sy _*variation influences cell subpopulation dynamics**. A color-code identical to that of Figure 2 panel C1 was defined for *P_sy _*variation (see Table 2), and model outputs were then consequently colored. Panels report the colored visualizations of subpopulations: A-CSCs, B-PCs, C-TCs, and D-DCs. Results revealed that *P_sy _*is responsible for a switching-like behavior, which discriminates between tumorigenesis (red and green lines) and unsustainable tumor growth (black lines). Output data are expressed using a logarithmic scale to reduce their wide range to a more manageable size.

We found that *P_sy _*is responsible for a switching-like behavior, which discriminates between tumorigenesis and unsustainable tumor growth. Specifically, as shown in Figure 10, *P_sy _*values smaller than 0.025 mainly lead to the death of the CSCs and of all the remaining tumor cells. On the other hand, when the probability of a symmetric division is slightly increased, a dynamic homeostasis starts to appear where cell birth and death are balanced and tumor size, after a first transitory phase, remains relatively constant. More precisely, the general behaviors that we observed are: (i) for *low P_sy _*values (black lines), i.e. *P_sy _*∈ [0, 0.25], tumor mainly does not grow; (ii) for *high P_sy _*values (red and green lines), i.e. *P_sy _*∈ (0.5, 1], populations grow until a plateau is reached and then maintained; and (iii) for *intermediate P_sy _*values (blue lines), i.e. *P_sy _*∈ (0.25, 0.5], both scenarios are possible.

**Figure 11:**
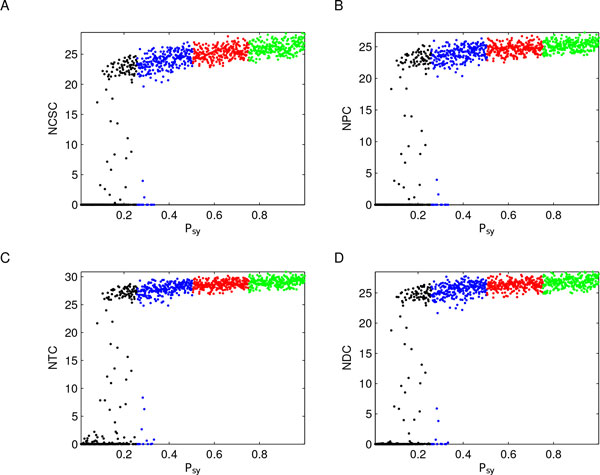
**Scatter plots of subpopulations and *P_sy _*variation**. Colored scatter plots of subpopulations at time *t *= 200 versus *P_sy _*variation remark that CSC symmetric proliferation is responsible of the two possible tumor scenarios. Specifically: black points are mainly associated with the unsustainable tumor growth, while green and red points correspond to tumorigenesis. Moreover, when *P_sy _*takes values in (0.25, 0.5], i.e. blue points, the switching is less clear, suggesting that other parameters might influence the system behavior in these cases.

**Figure 12:**
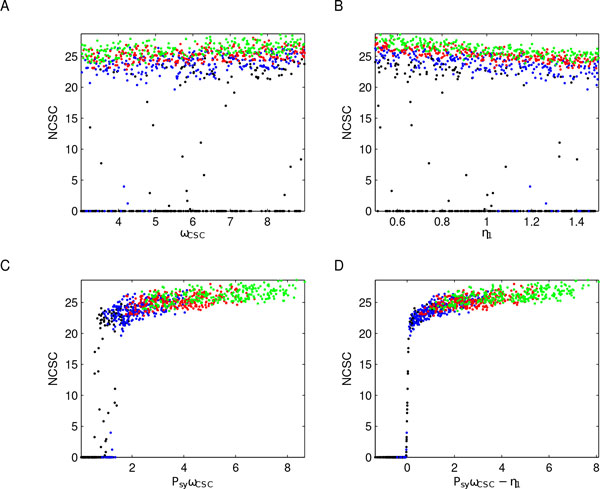
**Switching characterization**. Scatter plots of CSCs at time *t *= 200 were produced considering also the other key parameters and two *ad hoc *combinations of them. Specifically: panel A reports CSCs versus *ω_CSC _*, panel B refers to CSCs versus *η*_1_, panel C shows CSCs versus *P_sy _ω_CSC _*, and panel D displays CSCs versus *P_sy _ω_CSC _− η*_1_. All plots are colored using the *P_sy _*color-code previously defined, and they confirm the central role of this parameter. Indeed, *ω_CSC _*and *η*_1 _variations considered individually are not able to characterize the switch. On the other hand, combining these parameters with *P_sy _*it is possible to better characterize the switch when *P_sy _*assumes intermediate values.

A further characterization of different tumor evolution was provided by the scatter plot analysis, focusing on system dynamics at the end of the time interval, i.e. at time *t *= 200. In particular, Figure 11 shows subpopulation versus *P_sy _*values, where the points representing the outputs are colored in accordance with *P_sy _*levels. These results remark how CSC symmetric proliferation is responsible for a switching-like behavior in tumor evolution: black points are mainly associated with the unsustainable tumor growth scenario, while green and red points correspond to tumorigenesis, suggesting something akin to a putative *phenotypic tumor suppressor *function for this parameter. However, when *P_sy _*takes values in (0.25, 0.5] (blue points) the switching is less clear, suggesting that other parameters might influence the system behaviors in these cases. Therefore, starting from the results of the previous PRCC analysis on key parameters, we investigated also the roles of *ω_CSC _*and *η*_1 _in tumor evolution. Specifically, we produced scatter plots of subpopulations values versus both these parameters considered individually, and versus two combinations of them, namely *P_sy _∗ ω_CSC _*and *P_sy _∗ ω_CSC _− η*_1_, see Figure 12. These combinations were suggested by equations (1) and by the PRCC analysis that defined which type of monotonic relationships (positive/negative) key parameters had with model outputs. The results of these additional investigations confirmed our previous hypothesis that, when *P_sy _*assumes intermediate values, other factors - as the rate of cell division and differentiation - influence tumor evolution. More precisely, comparing Figure 11, panel A, with Figure 12, panels A and B, the central role of *P_sy _*is further emphasized being *ω_CSC _*and *η*_1 _variations considered individually not able to characterize the switch. Indeed, for each value of *ω_CSC _*or *η*_1 _it is possible to find CSCs in both configurations (low and high), while the two CSC states are well characterized by *P_sy _*values: black-blue points mark the low CSC concentrations, red-green points the high ones. On the other hand, combining these parameters with *P_sy _*it is possible to better characterize the switch when *P_sy _*assumes intermediate values. Indeed, comparing Figure 12 panels C and D with Figure 11 panel A there is a restriction of the *transition *zone between CSC concentrations. Notice that the strong influence of *P_sy _*is remarked one more time since red and green points correspond only to tumor growth, black points mainly define the unsustainable tumor growth scenario, while blue points can be found in both cases.

Summarizing, these results suggested a critical CSC symmetric proliferation value at which tumor stabilization occurs. Moreover, we identified an intermediate region in which other phenotype parameters involved in CSC variation cause tumor growth and maintenance.

**Figure 13:**
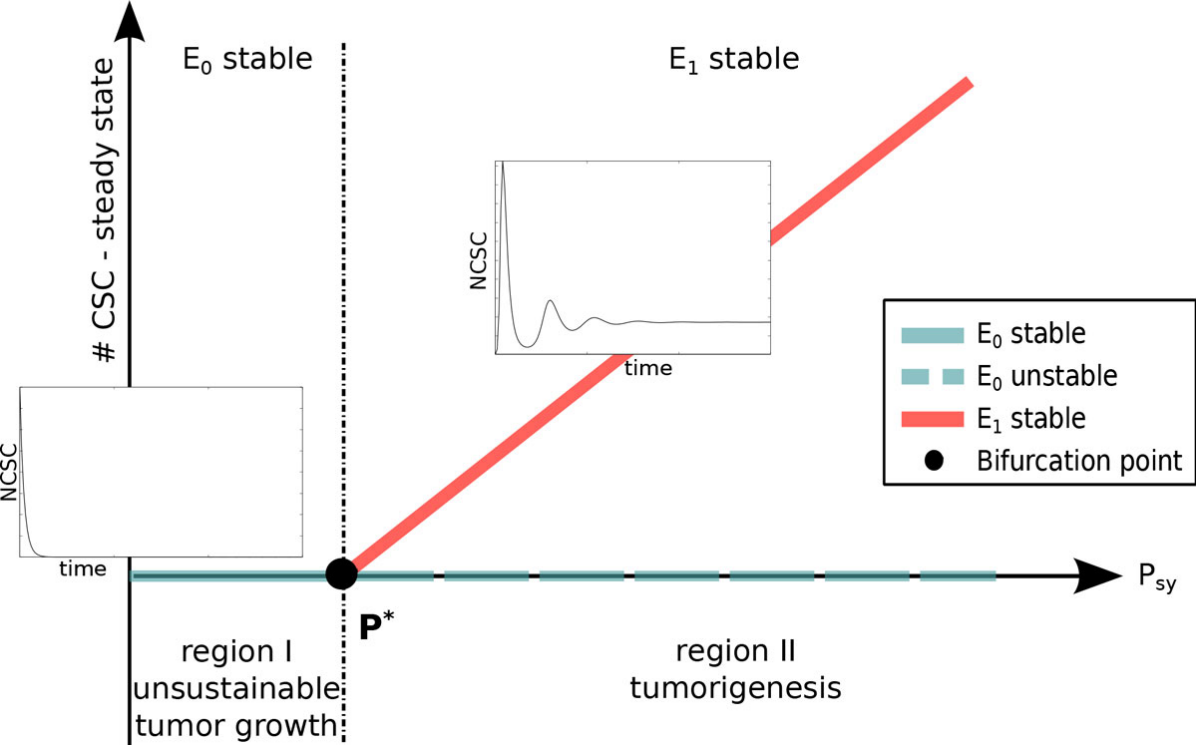
***P_sy _*bifurcation plots and CSC dynamics**. Bifurcation analysis on model (1) revealed that the system undergoes a transcritical bifurcation as *P_sy _*varies. Two steady states exist: *E*_0 _(trivial state) and *E*_1 _(positive state). The former one correspond to an unsustainable tumor growth, while the latter describes tumor growth and stabilization. When *P_sy _*reaches the transcritical bifurcation point *P_∗_, E*_0 _and *E*_1 _collide and interchange their stability conditions.

Starting from these results we performed a bifurcation analysis on system (1) and we found that the system undergoes a transcritical bifurcation as *P_sy _*varies. Figure 13 summarizes these results, showing how different tumor scenarios are associated with changes in *P_sy _*values. In details, two steady states exist: (i) the trivial one (*E*_0_), in which tumor does not grow; and (ii) a positive one (*E*_1_), in which tumor grows and then stabilizes. Varying *P_sy _, E*_0 _and *E*_1 _collide and interchanges their stability when the transcritical bifurcation point is reached. Specifically, studying the Jacobian matrix associated with system (1) it resulted that *E*_0 _is stable for $P_{sy}<P^{*}=\frac{\eta_{1}+\delta_{1}}{\omega_{CSC}}$. From a biological perspective, this threshold can be defined as the tumor *invasion boundary*. Indeed, for *P_sy _*<*P *^∗ ^the stable equilibrium is *E*_0_, which means that tumor cell do not survive. In the other case subpopulations can be always maintained, leading to tumor growth and stabilization.

## Conclusions

In this paper we have proposed a new work-flow that helps to characterize ODE system behaviors and to identify those parameters which mostly influence such behaviors. Our work-flow uses state of the art methodologies for sensitivity analysis, re-adapted to allow an easy identification, interpretation, and visualization of key model parameters.

When a limited amount of data is available, proper numerical estimates of model parameters are difficult to obtain. Estimated values must therefore be regarded as preliminary information, and alternative strategies must be identified to assess the quality of the results produced by the models. In these cases, our methodology can be followed to investigate the nature of the relationships between input parameters and output values. In particular, when parameters are difficult to be experimentally measured and may thus be affected by large variations, our methodology allows to study model behavior as parameters are varied with statistical techniques. By varying experimental settings, such as the distribution used, the parameter variation ranges, and their partition, our methodology allows to deeply investigate all system behaviors and to identify which parameters are relevant for explaining interesting output dynamics with a limited computational effort.

Having identified a restricted number of parameters which are relevant for the model, analytical approaches can then be applied within this simplified context. Our work-flow is a practical and informative tool to approach the study of complex systems such as the biological models where metabolic pathways are described or where detailed kinetics need to be accounted for.

The effectiveness of this methodology has been verified on two well-known models, whose results published in the literature have been accurately reproduced using our approach. After this preliminary validation, our methodology has been applied to a CSC-tumor model which extends a previous representation of the same phenomenon that we have published in [18] and that aims to figure out which are the phenotypic parameters that drive cancer growth.

Our CSC-tumor based model describes all phases of cancer progression and accounts for the negative feedback which TC subpopulation has on the proliferation rates of CSCs and PC1s. With the introduction of this feedback, we have been able to model cell auto growth limitation which has been demonstrated not only on stem cells during organogenesis [32], but also in cancer cells during tumor growth [33]. Applying our methodology to this model we found that the probability of CSC symmetric proliferation is responsible for a switching-like behavior. Specifically, *P_sy _*discriminates between two possible scenarios: tumorigenesis and unsustainable tumor growth. More precisely, if CSC symmetric proliferation probability has low values, then the system falls in the non-growth scenario. Otherwise, for high *P_sy _*values, the only possibility is the tumor growth, and no other parameters considered individually are able to characterize the switch. Notice that the condition identifying the non growth scenario is a *relaxed *condition since the choice *r *= 4 did not allow us to unequivocally discriminate the threshold among tumorigenesis and unsustainable tumor growth. However, this initial analysis revealed the existence of such a threshold, that we had afterwards investigated in depth by means of bifurcation analysis. Moreover, we found that there is a transition zone in which it is necessary to consider together CSC symmetric proliferation, CSC proliferation rate, and CSC differentiation rate, in order to precisely characterize the tumor evolution. This supports the notion that CSC phenotypic plasticity is able to lead to functionally distinct cancer subpopulations that support and modulate the overall tumor growth and maintenance [2]. Moreover, our finding has been supported by experimental evidences suggesting that the deregulation of asymmetric proliferation contributes to cancer initiation [34,35].

All dynamics considered in our phenomenological mathematical model are related to interactions among cell populations and are based on cancer stem cell fate, including cell proliferation, differentiation and death. These mechanisms are expressed through the phenotypic parameters of the model, which provides a global description of the tumor growth. However, a deeper characterization of this phenomenon might consider also: (i) the intrinsic noise present in biological data, and (i) the cellular processes within cancer stem cells which control cell fate, and which are tagged as hallmark of cancers. Examples of these processes are the unfolded protein response [36] and the autophagy [37], which are stress response phenomena and which modulate tumor microenvironment, leading to metabolic reprogramming and changes in cancer stem cells fate.

A more detailed investigation could be conducted - as a future work - integrating in our model both the noise and those internal cellular mechanisms which control cancer stem cell fate, such that it might be possible to better characterize which are the microenvironment stimuli that mostly influence symmetric proliferation decision. Results presented in this paper could hence be used to facilitate and improve this integration. Indeed, following the idea presented in our recent paper on multi-level modeling [38], our population model (1) could be corroborated by an additional level focusing on cellular internal dynamics.

## Competing interests

The authors declare that they have no competing interests.

## Authors' contributions

CF, GB, FC, and MB designed the model and methodology. SMH, OBR, and ARA supervised the work-flow definition. CF performed in-silico experiments. FC and MB supervised the project. CF, GB, RAC, FC, and MB wrote the manuscript. All authors read and approved the final manuscript.

## Supplementary Material

Additional File 1 CELL CYCLE -- Parameter values of cell cycle model described in [28]. Click here for file

Additional File 2 OUTPUTS OF CELL CYCLE MODEL -- Model solutions were calculated for each parameter combination over the time interval *T *= [0, 50], and are here reported for CycE (panel A) and RB (panel B). The bimodal behavior of CycE results evident from these experiments. Click here for file

Additional File 3 PRCC ANALYSIS OF CELL CYCLE MODEL -- PRCCs of input/output data revealed serum as the key parameter. Results relative to CycE and RB during the whole time interval are reported in panels A and B, respectively. Yellow areas represent the zones of non-significant PRCC values, while blue lines correspond to serum PRCC values. The strong positive (negative) monotonic relationship between serum and CycE (RB) is remarked, being the blue line close to 1 (-1) in the whole interval and *isolated *from the other PRCC values; see panel A (panel B). Click here for file

Additional File 4 SERUM VARIATION INFLUENCES CELL CYCLE DYNAMICS -- A color-code identical to that of Figure 2 panel C1 was defined for serum variation, and model outputs were then consequently colored. Panels A and B report the colored visualizations of CycE and RB, respectively. RB levels decrease as serum increases: colors are well clustered and stratified in the order expressing a serum decrement (green-red-blue-black). CycE distribution, instead, exhibits a positive correlation and a bimodal dependence on serum concentration: low CycE state is mainly characterized by black and blue lines (low serum concentration), while the high state corresponds to green lines (high serum concentration). Click here for file

Additional File 5 SCATTER PLOTS OF CELL CYCLE AND SERUM VARIATION -- Colored scatter plots of model variables at time *t *= 50 versus serum variations were produced, and are here reported for CycE and RB in panles A and B respectively. This graphs show CycE and RB configurations at equilibrium, further emphasizing the role of serum variation. In panel A the low CycE state is characterized by black-blue points (low serum), while the high state by red-green points (high serum), thus revealing that CycE distribution exhibits bimodal dependence on serum concentration. In panel B, instead, RB levels decrease with serum concentration making evident the negative monotonic relationship between serum and RB. Click here for file

Additional File 6 APOPTOSIS -- Parameter values of apoptosis model described in [28]. Click here for file

Additional File 7 OUTPUTS OF APOPTOSIS MODEL -- Model solutions were calculated for each parameter combination over the time interval *T *= [0, 500], and are here reported for BAXmT (panel A), BH3 (panel B), and BCL2 (panel C). The bimodal behavior of BAXmT results evident from these experiments. Click here for file

Additional File 8 STRESS AND BCL2T CONCENTRATION INFLUENCE APOPTOSIS DYNAMICS -- A color-code identical to that of Figure 2 panel C1 was assigned to both stress and BCL2T variations, and model variables (i.e. BAXmT, BH3, and BCL2T) were then colored in accordance to these color-codes. Specifically, panels A, B, and C refer to the stress coloration, while panels D, E, and F refer to the BCL2T one. Click here for file

Additional File 9 PRCC ANALYSIS OF APOPTOSIS MODEL -- PRCCs of input/output data revealed stress and BCL2T as the key parameters. Results relative to BAXmT, BH3, and BCL2 during the whole time interval are reported in panels A, B, and C, respectively. Yellow areas represent the zones of non-significant PRCC values, while blue lines correspond to stress PRCC values, and red ones refer to those of BCL2T. Click here for file

Additional File 10 CELL POPULATIONS -- Parameter values of cell population model (1). Click here for file

## Declarations

Publication of the article was funded by grants from the Epigenomics Flagship Project EPIGEN, MIUR- CNR, European Seventh framework program, Health.2012.1.2-1, NGS-PTL grant n. 306242, and by project grant Nr. 10-15-1432/HICI from the King Abdulaziz University of Saudi Arabia.

This article has been published as part of *BMC Systems Biology *Volume 9 Supplement 3, 2015: Proceedings of the Italian Society of Bioinformatics (BITS): Annual Meeting 2014: Systems Biology. The full contents of the supplement are available online at http://www.biomedcentral.com/bmcsystbiol/supplements/9/S3.

## References

[B1] Anderson A Quaranta V Integrative matheamtical oncology Nat Reviews Cancer 2008 8 227 234 10.1038/nrc2329 18273038

[B2] Hanahan D Weinberg R Hallmarks of cancer: the next generation Cell 2011 144 646 674 10.1016/j.cell.2011.02.013 21376230

[B3] Hanahan D Weinberg R The hallmarks of cancer Cell 2000 100 57 70 10.1016/S0092-8674(00)81683-9 10647931

[B4] Conti L Ruiu R Barutello G Macagno M Bandini S Cavallo F Lanzardo S Microenvironment, oncoantigens, and antitumor vaccination: lessons learned from balb-neut mice Hindawi Publishing Corporation BioMed Research International 2014 10.1155/2014/534969 PMC4065702 25136593

[B5] Frank N Schatton T Frank M The therapeutic promise of the cancer stem cell concept The journal of clinical investigation 2010 120 10.1172/JCI41004 PMC2798700 20051635

[B6] Cho R Clarke M Recent advances in cancer stem cells Curr Opin Genet Dev 2008 18 10.1016/j.gde.2008.01.017 18356041

[B7] Ho MM Ng AV Lam S Hung JY Side population in human lung cancer cell lines and tumors is enriched with stem-like cancer cells Cancer Research 2007 67 10.1158/0008-5472.CAN-06-3557 17510412

[B8] Singh SK Hawkins C Clarke ID Squire JA Bayani J Hide T Identification of human brain tumour initiating cells Nature 2004 432 10.1038/nature03128 15549107

[B9] O'Brien CA Pollett A Gallinger S Dick JE A human colon cancer cell capable of initiating tumour growth in immunodeficient mice Nature 2007 445 10.1038/nature05372 17122772

[B10] Marciniak-Czochra A Stiehl T Ho A Jager W Wagner W Modeling of asymmetric cell division in hematopoietic stem cells - regulation of self-renewals is essential for efficient repopulation Stem Cells and development 2009 18 10.1089/scd.2008.0143 18752377

[B11] Mirams G Fletcher A Maini P Byrne H A theoretical investigation of the effect of proliferation and adhesion on monoclonal conversion in the colonic crypt Journal of Theoretical Biology 2013 10.1016/j.jtbi.2012.08.002 22902425

[B12] Dingli D Traulsen A Michor F (a)symmetric stem cell replication and cancer PLoS Comp Bio 2007 3 10.1371/journal.pcbi.0030053 PMC1828703 17367205

[B13] Gupta P Fillmore C Jiang G Shapira S Tao K Kuperwasser C Lander E Stochastic state transitions give rise to phenotypic equilibrium in populations of cancer cells Cell 2011 146 633 644 10.1016/j.cell.2011.07.026 21854987

[B14] Nguyen L Vanner R Dirks P Eeves C Cancer stem cells: an evolving concept Nature Review Cancer 12 2012 10.1038/nrc3184 22237392

[B15] Liu X Johnson S Liu S Kanojia D Yue W Singh U Wang Q Wang Q Nie Q Chen H Nonlinear growth kinetics of breast cancer stem cells: Implications for cancer stem cell targeted therapy Sci Rep 2013 3 10.1038/srep02473 PMC3747506 23959163

[B16] Scott J Hjelmeland A Chinnaiyan P Anderson A D B Microenvironmental variables must influence intrinsic phenotypic parameters of cancer stem cells to affect tumourigenicity PLOS Compultational Biology 2014 10 10.1371/journal.pcbi.1003433 PMC3894166 24453958

[B17] Youssefpour H Li X A L Lowengrub J Multispecies model of cell lineages and feedback control in solid tumors Journal of theoretical biology 2012 304 10.1016/j.jtbi.2012.02.030 PMC3436435 22554945

[B18] Fornari C Beccuti M Lanzardo S Conti L Balbo G Cavallo F Calogero R Cordero F A mathematical-biological joint effort to investigate the tumor-initiating ability of cancer stem cells PLOSE One 2014 9 10.1371/journal.pone.0106193 PMC4153566 25184361

[B19] Marino S Hogue I Ray C Kirschner D A methodology for performing global uncertainity and sensitivity analysis in systems biology Journal of Theoretical Biology 2008 254 178 196 10.1016/j.jtbi.2008.04.011 18572196 PMC2570191

[B20] Lo W Chou C Gokoffski FK Wan Lander A Calof QA Nie Feedback regulation in multistage cell lineages Mathematical Biosciences and Engineering 2009 6 10.3934/mbe.2009.6.59 PMC2756546 19292508

[B21] Murray J Mathematical Biology: I. An Introduction (Interdisciplinary Applied Mathematics) 2007 3

[B22] Saltelli A Chan K Scott EM Sensitivity Analysis 2009

[B23] Helton J Davis F Sampling-based Methods for Uncertainty and Sensitivity Analysis 2000 10.1111/0272-4332.00041 12088236

[B24] Iman R Helton J An investigation of uncertainty and sensitivity analysis techniques for computer models Risk Anal 1988 8 71 90 10.1111/j.1539-6924.1988.tb01155.x

[B25] Mckay M Beckman R Conover W Comparison of 3 methods for selecting values of input variables in the analysis of output from a computer code Technometrics 1979 21 239 245

[B26] Helton J Davis F Latin hypercube sampling and the propagation of uncertainty in analyses of complex systems Reliab Eng Syst Saf 2003 81 23 69 10.1016/S0951-8320(03)00058-9

[B27] Anderson T An Introduction to Multivariate Statistical Analysis, third ed. edn. Wiley Series in Probability and Statistics 2003 Wiley-Interscience, Hoboken, NJ

[B28] Tyson J Baumann T Chen C Verdugo I Tabassoly Y Wang Y Weiner L Clarke R Dynamic modelling of oestrogen signalling and cell fate in breast cancer cells Nature Reviews Cancer 2010 11 523 532 10.1038/nrn2850 PMC3294292 21677677

[B29] Turner C Kohandel M Investigating the link between epithelial-mesenchymal transition and the cancer stem cell phenotype: A mathematical approach Journal of Theoretical Biology 2010 265 329 335 10.1016/j.jtbi.2010.05.024 20648969

[B30] Molina-Pena R Álvarez M A simple mathematical model based on the cancer stem cell hypothesis suggests kinetic commonalities in solid tumor growth Plos One 2012 7 10.1371/journal.pone.0026233 PMC3281810 22363395

[B31] Conti L Lanzardo S Arigoni M Antonazzo R Radaelli E Cantarella D Calogero R Cavallo F The noninflammatory role of high mobility group box 1/toll-like receptor 2 axis in the self-renewal of mammary cancer stem cells FASEB Journal 2013 10 10.1096/fj.13-230201 23970797

[B32] Johnston M Edwards C Bodmer W Maini P Chapman S Mathematical modeling of cell population dynamics in the colonic crypt and in colorectal cancer Proc Natl Acad Sci USA 2007 104 4008 4013 10.1073/pnas.0611179104 17360468 PMC1820699

[B33] Rodriguez-Brenes I Komarova N Wodarz D Evolutionary dynamics of feedback escape and the development of stem-cell-driven cancers Proc Natl Acad Sci USA 2011 108 18983 18988 10.1073/pnas.1107621108 22084071 PMC3223454

[B34] Sugiarto S symmetry-defective oligodendrocyte progenitors are glioma precursors Cancer Cell 2011 20 328 340 10.1016/j.ccr.2011.08.011 21907924 PMC3297490

[B35] Ito T Regulation of myeloid leukaemia by the cell-fate determinant musashi Nature 2010 466 765 768 10.1038/nature09171 20639863 PMC2918284

[B36] Erguler K Pieri M Deltas C A mathematical model of the unfolded protein stress response reveals the decision mechanism for recovery, adaptation and apoptosis BMC Systems Biology 2013 7 10.1186/1752-0509-7-16 PMC3695880 23433609

[B37] Tavassoly I Shajahan A Parmar J Baumann W Clarke R Tyson J Dynamical modeling of the interaction between autophagy and apoptosis in mammalian cells: a systems pharmacology framework arXiv preprint 2013 10.1002/psp4.29 PMC4429580 26225250

[B38] Cordero F Beccuti M Fornari C Lanzardo S Conti L Federica C Balbo G Calogero R Multi-level model for the investigation of oncoantigen-driven vaccination effect BMC Bioinformatics 2013 14 10.1186/1471-2105-14-S6-S11 PMC3633011 23734974

