# Supplementary figures and images for: A versatile mathematical work-flow to explore how Cancer Stem Cell fate influences tumor progression
Source: BMC Syst Biol. 2015 Jun 1;9(Suppl 3):S1. doi: 10.1186/1752-0509-9-S3-S1 (PMC4464028; doi:10.1186/1752-0509-9-S3-S1)

A

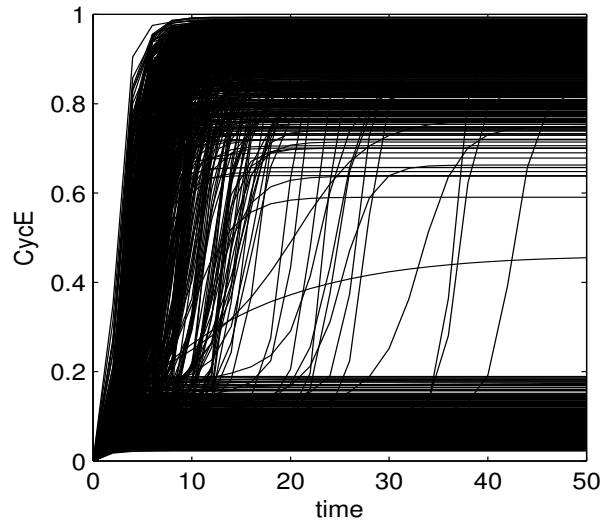

B

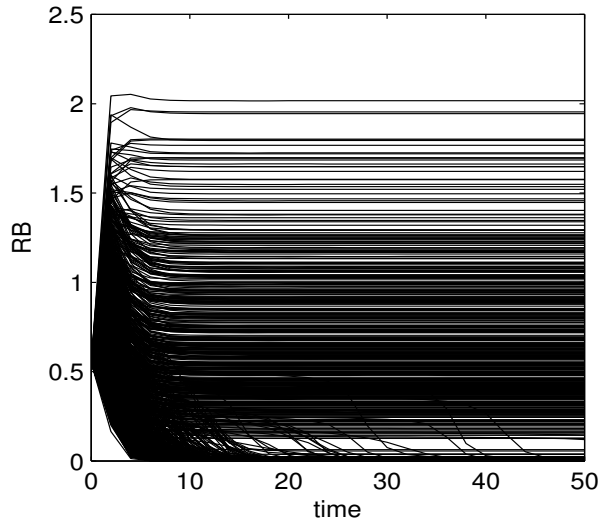

Supplement: Additional File 1 — CELL CYCLE -- Parameter values of cell cycle model described in [28]. [file 1752-0509-9-S3-S1-S1.pdf]

A

CycE

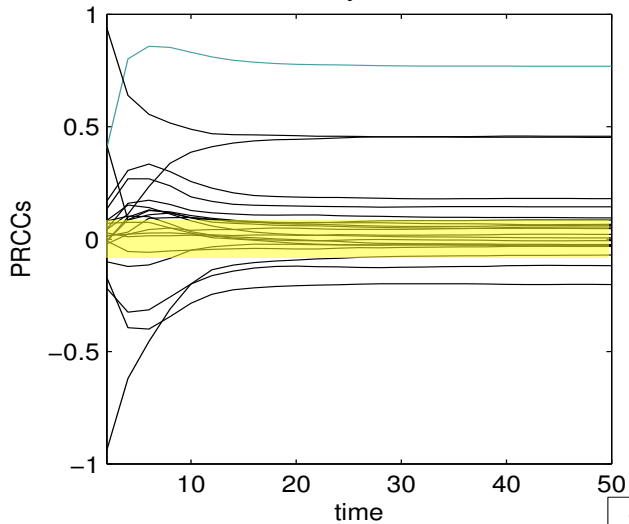

B

RB

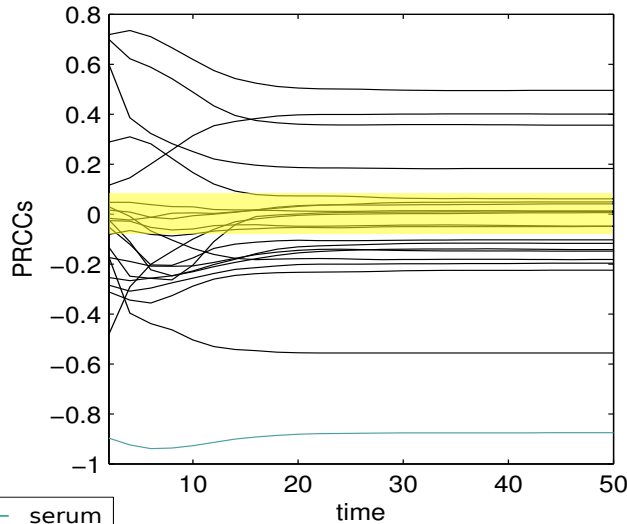

Supplement: Additional File 2 — OUTPUTS OF CELL CYCLE MODEL -- Model solutions were calculated for each parameter combination over the time interval T = [0, 50], and are here reported for CycE (panel A) and RB (panel B). The bimodal behavior of CycE results evident from these experiments. [file 1752-0509-9-S3-S1-S2.pdf]

A

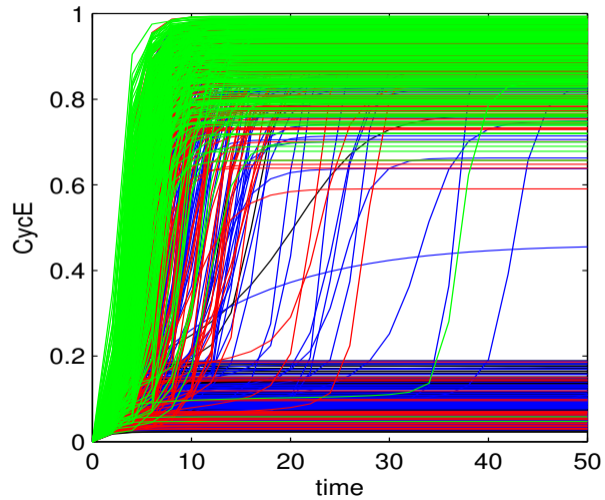

B

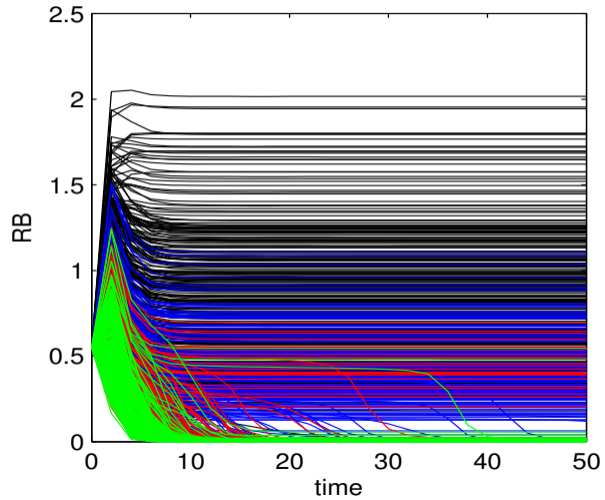

Supplement: Additional File 3 — PRCC ANALYSIS OF CELL CYCLE MODEL -- PRCCs of input/output data revealed serum as the key parameter. Results relative to CycE and RB during the whole time interval are reported in panels A and B, respectively. Yellow areas represent the zones of non-significant PRCC values, while blue lines correspond to serum PRCC values. The strong positive (negative) monotonic relationship between serum and CycE (RB) is remarked, being the blue line close to 1 (-1) in the whole interval and isolated from the other PRCC values; see panel A (panel B). [file 1752-0509-9-S3-S1-S3.pdf]

A

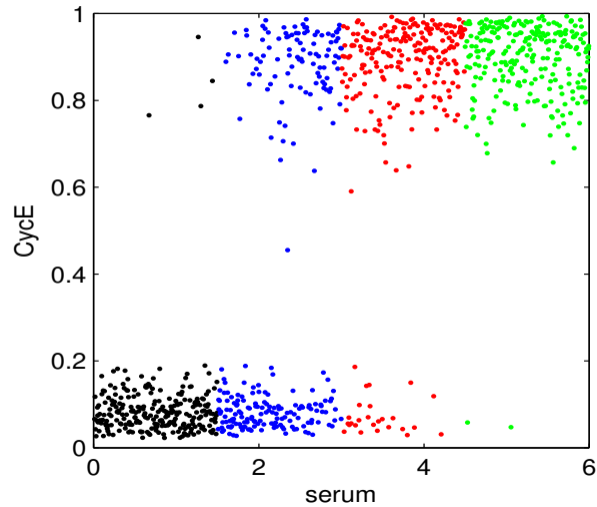

B

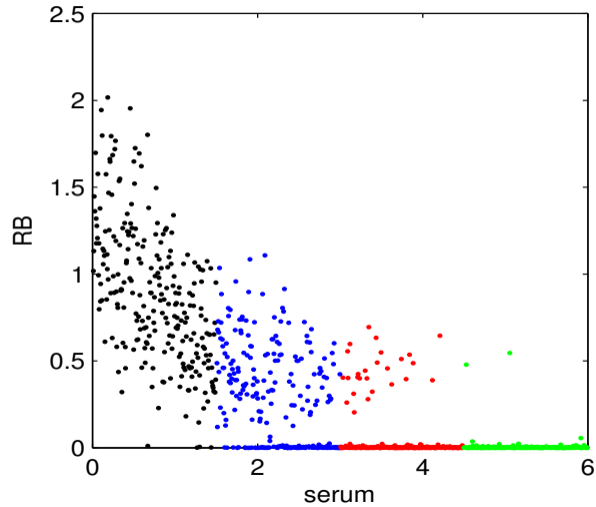

Supplement: Additional File 4 — SERUM VARIATION INFLUENCES CELL CYCLE DYNAMICS -- A color-code identical to that of Figure 2 panel C1 was defined for serum variation, and model outputs were then consequently colored. Panels A and B report the colored visualizations of CycE and RB, respectively. RB levels decrease as serum increases: colors are well clustered and stratified in the order expressing a serum decrement (green-red-blue-black). CycE distribution, instead, exhibits a positive correlation and a bimodal dependence on serum concentration: low CycE state is mainly characterized by black and blue lines (low serum concentration), while the high state corresponds to green lines (high serum concentration). [file 1752-0509-9-S3-S1-S4.pdf]

A

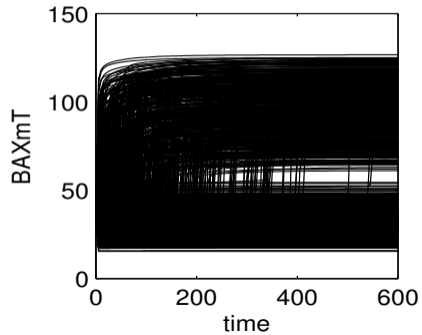

B

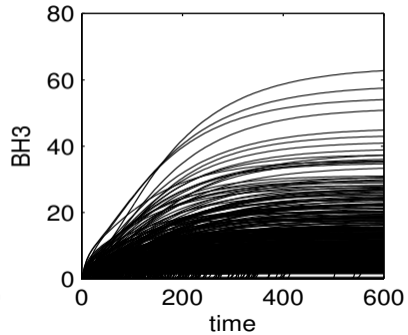

C

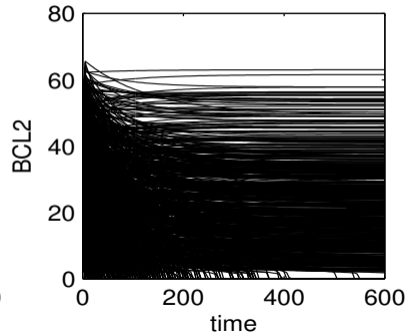

Supplement: Additional File 5 — SCATTER PLOTS OF CELL CYCLE AND SERUM VARIATION -- Colored scatter plots of model variables at time t = 50 versus serum variations were produced, and are here reported for CycE and RB in panles A and B respectively. This graphs show CycE and RB configurations at equilibrium, further emphasizing the role of serum variation. In panel A the low CycE state is characterized by black-blue points (low serum), while the high state by red-green points (high serum), thus revealing that CycE distribution exhibits bimodal dependence on serum concentration. In panel B, instead, RB levels decrease with serum concentration making evident the negative monotonic relationship between serum and RB. [file 1752-0509-9-S3-S1-S5.pdf]

A

BAXmT

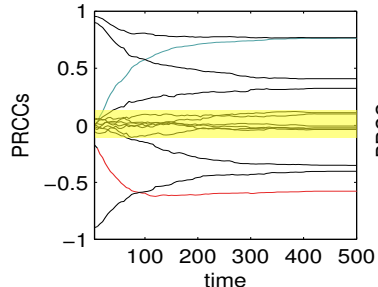

B

BH3

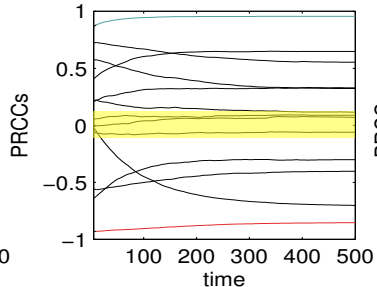

C

BCL2

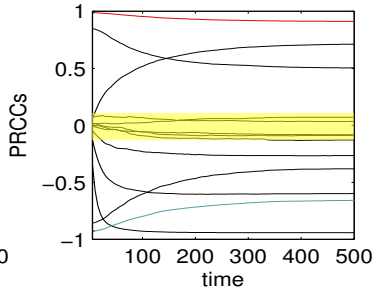

— stress  
— BCL2T

Supplement: Additional File 6 — APOPTOSIS -- Parameter values of apoptosis model described in [28]. [file 1752-0509-9-S3-S1-S6.pdf]

A

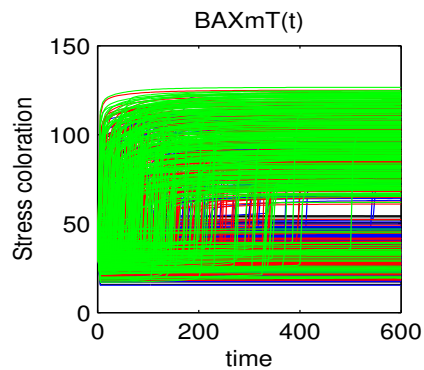

B

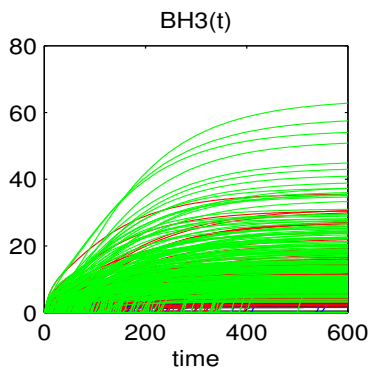

C

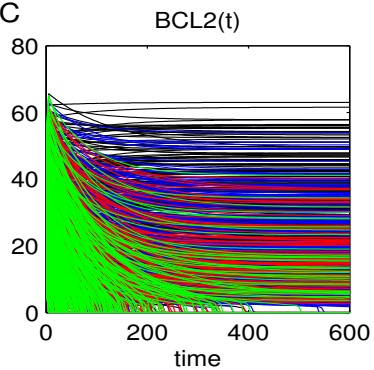

D

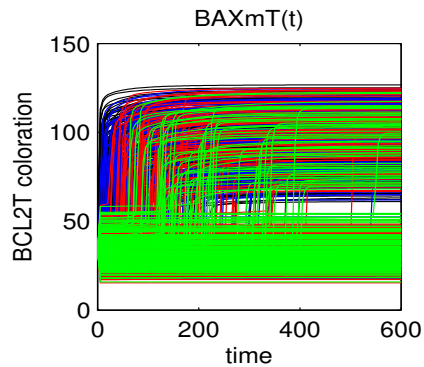

E

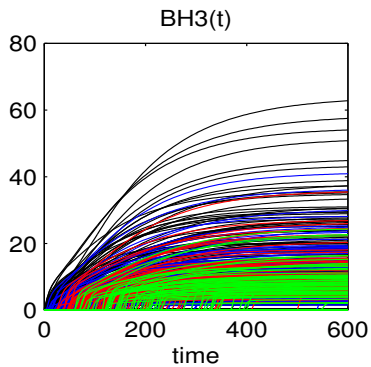

F

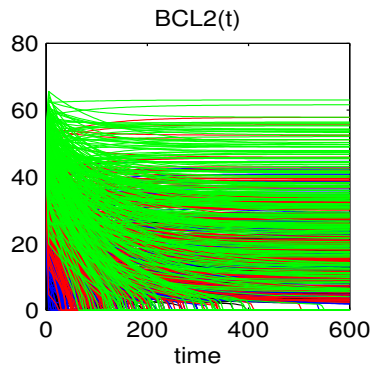

Supplement: Additional File 7 — OUTPUTS OF APOPTOSIS MODEL -- Model solutions were calculated for each parameter combination over the time interval T = [0, 500], and are here reported for BAXmT (panel A), BH3 (panel B), and BCL2 (panel C). The bimodal behavior of BAXmT results evident from these experiments. [file 1752-0509-9-S3-S1-S7.pdf]
